# Innovations in Practice: Brief behavioral parent training for children with impairing ADHD characteristics – a pilot study

**DOI:** 10.1111/camh.12743

**Published:** 2024-12-07

**Authors:** Marijn Nijboer, Roos van Doornik, Annabeth P. Groenman, Saskia van der Oord, Rianne Hornstra, Barbara van den Hoofdakker, Tycho J. Dekkers

**Affiliations:** ^1^ Accare Child Study Centre Groningen The Netherlands; ^2^ Department of Child and Adolescent Psychiatry University Medical Centre Groningen, University of Groningen Groningen The Netherlands; ^3^ Research Institute of Child Development and Education University of Amsterdam Amsterdam The Netherlands; ^4^ Clinical Psychology, Faculty of Psychology and Educational Sciences KU Leuven Leuven Belgium; ^5^ Department of Clinical Psychology and Experimental Psychopathology University of Groningen Groningen The Netherlands; ^6^ Levvel, Academic Centre for Child and Adolescent Psychiatry Amsterdam The Netherlands; ^7^ Department of Child and Adolescent Psychiatry Amsterdam University Medical Centres (AUMC) Amsterdam The Netherlands; ^8^ Department of Psychology University of Amsterdam Amsterdam The Netherlands

**Keywords:** ADHD, behavior problems, behavior therapy, parent training

## Abstract

**Background:**

Behavioral parent training (BPT) is a well‐established intervention for children with attention‐deficit/hyperactivity disorder (ADHD), but most programs are long, which may limit their accessibility. This could be improved by making programs shorter. Here, we studied (1) the feasibility of a new brief BPT program and its procedures, and (2) pre–post changes in daily rated problem behaviors (primary outcome), children's disruptive behaviors, ADHD/ODD characteristics, impairment, and parents' sense of parenting competence (secondary outcomes).

**Methods:**

We conducted a nonrandomized pilot study including parents of 28 children (4–12 years) with impaired ADHD characteristics. We examined treatment dropout, parent and therapist satisfaction, recruitment rates, study drop‐out, measurement response and completion rates, acceptability of measurements according to parents, and treatment fidelity. Pre–post changes in the treatment group were compared to those in a historical control group using mixed model analysis, except for those outcomes that were not assessed in the control group. Within‐group differences were analyzed for all outcomes.

**Results:**

Feasibility of the program and study procedures were good. Treatment dropout was 14.2%, parents and therapists were satisfied with the new program. We recruited 1.5 participants per month, study dropout was 10.7%, response/completion rates ranged from 82% to 100%, measurements were acceptable for parents, and treatment fidelity was 96%. We found substantial within‐group changes (*d*'s = .68–.77) and medium‐sized between‐group changes (*d*'s = .46–.48) on daily rated problem behaviors. We observed no changes on most of the secondary outcomes, except for disruptive behaviors and impairment.

**Conclusion:**

Our newly developed brief BPT program was feasible and we observed improvements in children's daily‐rated problem behaviors. These results suggest that brief BPT might be beneficial for clinical practice if the findings are confirmed in large‐scale randomized controlled trials.

**Key Practitioner Messages**

**What is known?**

- Most behavioral parent training (BPT) programs for ADHD are long, which may limit their accessibility.

**What is new?**

- We developed a new brief BPT program of three sessions, containing effective behavioral techniques.

**What is significant for clinical practice?**

- The brief BPT was rated highly by parents and therapists, and dropout was relatively low. Together, the high satisfaction of parents and therapists, low dropout rates, and reduction in children's behavioral problems suggest that brief BPT could potentially contribute to improving access to evidence‐based treatment for children with impairing ADHD characteristics.

## Introduction

Behavioral parent training (BPT) is a well‐established intervention for children with attention‐deficit/hyperactivity disorder (ADHD; Dekkers et al., 2022), but its accessibility for parents is suboptimal (Chacko et al., 2016). Most BPT programs include more than 10 sessions, and many parents experience practical barriers to organize this (Dekkers et al., 2022). Such practical barriers could be reduced by making BPT briefer, which could improve accessibility and consumer satisfaction. However, the evidence on the effects of brief BPT (<8 sessions) is limited (Tully & Hunt, 2016).

We recently conducted a microtrial into the effects of different behavioral techniques commonly used in BPT and showed that two 2‐h sessions of either stimulus‐control or contingency management techniques were more effective than waitlist in reducing daily‐rated problem behaviors in children with ADHD (Hornstra et al., 2021). Moreover, we found similar effect sizes as in full parenting interventions (Hornstra et al., 2022). This encouraged us to further develop a brief BPT program, including the stimulus‐control and contingency management techniques that were effective in the microtrial.

This pilot study was conducted in preparation of a randomized controlled trial (RCT). Our first objective was to evaluate (a) the feasibility of the program by assessing treatment dropout and parent and therapist satisfaction and (b) the feasibility of study procedures by examining recruitment rates, study dropout, measurement response/completion rates, acceptability of measurements according to parents, and treatment fidelity. Our second objective was to assess pre–post changes in the treatment group compared to historical controls on daily‐rated problem behavior (primary outcome) and children's disruptive behavior, ADHD/ODD characteristics, impairment, and parents' sense of parenting competence (secondary outcomes).

## Method

### Participants and procedure

We recruited parents between April 2021 and October 2022 through a clinical team, specialized in working with children with externalizing problems, as part of an outpatient mental health clinic for children from 0 to 18 years in the Netherlands. Parents were informed about the study by their clinician and, when interested, researchers provided information before obtaining informed consent. We included parents of children ages 4–12, attending primary education with an IQ above 70. Children had at least four impairing ADHD characteristics at home and at least two at school, measured by the Parent Interview for Child Symptoms (PICS; Schachar, Ickowicz, & Sugarman, 2000) and the Teacher Telephone Interview (TTI; Tannock, Hum, Masellis, Humphries, & Schachar, 2002).

Participants were excluded if (a) children were currently using or had used psychotropic medication in the past month (because these medications may influence our outcome measures such as ADHD symptoms and behavioral problems); (b) children had a diagnosis of autism spectrum disorder reported in the medical file (because these children and their parents might need other interventions); (c) the family experienced problems that required immediate intervention, for example, crisis (because the training is not a crisis intervention); (d) parents received BPT in the last year (because comparable techniques were taught to these parents already); (e) the child was not living in the same household during weekdays (ensuring that the parent who received BPT could practice the intervention plans sufficiently). These criteria also applied to the historical control group.

The study was preregistered (ClinicalTrials.gov ID: NCT05452954) and the Medical Ethics Review Board of the University Medical Centre Groningen (UMCG) approved that it did not meet the conditions of the Medical Research Involving Human Subjects Act (WMO). The study complied with the declaration of Helsinki (1964) and its later revisions.

### Design

**Figure 1:**
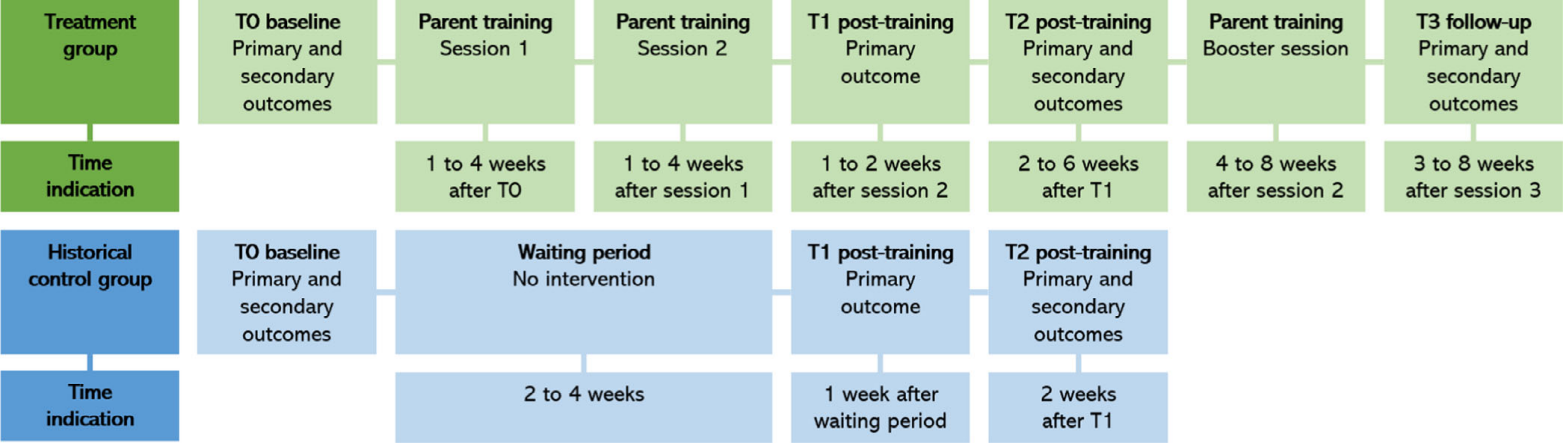
Overview of sessions and measurements

We conducted a nonrandomized historically controlled pilot study, comparing pre–post changes in the treatment group with a historical control group. The historical control group consisted of the parents in the waitlist condition from our previous study with an almost identical design (Hornstra et al., 2021). For the current pilot study, we added a third (booster) session to the intervention and a follow‐up measurement (T3). For an overview of sessions and measurements, see Figure 1. The timing of measurements and sessions could differ per family to optimize flexibility in planning the sessions.

### Treatment

The intervention (PAINT‐P: Psychosocial ADHD and behavioral problems INTerventions—Parent training) consisted of two 2‐h session and a 1‐h booster session. Licensed behavioral therapists who were experienced in applying BPT in the context of clinical studies were invited to participate. Therapists received supervision on study procedures every other week from the first author.

Before the intervention, parents selected four problem behaviors (see Van den Hoofdakker et al., 2007) in specific situations, of which two were targeted in the intervention. In the first session, the therapist provided brief psychoeducation about inhibition, working memory and motivation in children with ADHD and made a functional analysis of the first targeted behavior. This analysis included a systematic examination of the antecedents and consequences of the problematic behavior, to better understand why the behavior was occurring. Based on this, the therapist and parents made an intervention plan, including stimulus‐control techniques (giving instructions, setting rules, structuring the environment, and anticipating problematic behaviors) and praise. This plan was practiced in the session and parents were instructed to carry it out every day. In the second session, the first plan was evaluated and the therapist conducted a functional analysis of the second targeted behavior. Subsequently, therapist and parents made an intervention plan including both stimulus‐control and contingency management techniques (reinforcement of positive behavior, ignoring negative behavior, and setting mild negative consequences). Again, the intervention plan was practiced and parents were instructed to carry out both action plans on a daily basis. In the booster session, the therapist and parents adjusted the intervention plans if necessary and the therapist encouraged parents to persevere and generalize the techniques to other behaviors and situations.

### Measures

#### Feasibility

Feasibility measures are shown in Table 1. Appendix S1 provides more details on these measures.

**Table 1:** Feasibility measures

Feasibility	Measured by
*Treatment program*	
Treatment dropout	% Parents dropping out of treatment before finishing the booster session
Parent satisfaction	Self‐developed online questionnaire (25 items)
Therapist satisfaction	Self‐developed online questionnaire (18 items)
*Study procedures*	
Recruitment	Number of inclusions per month, number of parents not willing to participate
Study dropout	% Parents dropping out of measurements before finishing follow‐up measurement
Measurement response/completion rates	% of responded and completed questionnaires
Acceptability of measurements	Self‐developed online questionnaire (7 items)
Treatment fidelity	% Addressed session content according to therapists and independent coders

#### Pre–post changes

Our primary outcome consisted of the mean severity of daily ratings of the four individual target behaviors in specific situations. We assessed these ratings through daily phone calls with parents. Secondary outcomes were the Intensity subscale of the Eyberg Child Behavior Inventory (ECBI; Eyberg & Pincus, 1999), the Inattention and Hyperactivity/Impulsivity subscales of the Strengths and Weaknesses of ADHD symptoms and Normal Behavior Rating Scale (SWAN, Swanson, Schuck, & Porter, 2012), the oppositional defiant disorder (ODD) subscale of the Dutch Disruptive Behavior Disorder Rating Scale (DBDRS, Oosterlaan et al., 2008), the Impairment Rating Scale (IRS, Fabiano et al., 2006), and the Efficacy subscale of the Parenting Sense of Competence Scale (PSOC, Gibaud‐Wallston & Wandersman, 1978). These questionnaires were completed online (see Appendix S1 for more information).

### Data analysis

A power analysis was conducted for the pre–post within‐group analysis. Based on the lowest within‐group effect size in our previous study (*d* = .57; Hornstra et al., 2021), an effect size of *f* = .285 (*f* = *d*/2) was assumed. Given α = .05 and 1−β = .80, we required 28 participants in our treatment condition.

We analyzed outcome data on an intention‐to‐treat basis. Within‐group changes and between‐group differences were investigated with mixed model analysis in SPSS (version 29), with outcomes (level 1) nested within participants (level 2), within therapists (level 3), and time (T0, T1, T2, and T3) as a fixed factor. For the between‐group analyses, condition was added as a fixed factor. We included a random intercept at the therapist level only if the likelihood ratio test showed a significant improvement of the model fit. We used maximum likelihood estimation to accurately estimate our outcome with little missing data. For effect sizes, we calculated Cohen's *d* with the estimated marginal means and pooled standard deviations for each specific group and timepoint. For within‐group comparisons, Cohen's *d* represents comparisons between timepoints. For between‐group comparisons, Cohen's *d* represents comparisons between groups at one timepoint.

Finally, we conducted a post hoc correlation analysis to assess whether the number of days between T0 and T3 was associated with the difference between T0 and T1, T2 and T3, respectively, on our primary outcome.

## Results

### Descriptives

Participant characteristics are shown in Table 2. At baseline, groups differed in age and ADHD presentation. See Appendix S2 (Table S1 and Figure S2) for descriptive statistics on all outcomes.

**Table 2:** Participant characteristics

	Treatment group (*n* = 28)	Historical control group (*n* = 30)	Comparison *t* (df)/χ^2^ (df)
*Demographics*			
Age in years, *M* (*SD*)	8.82 (1.87)	7.73 (1.76)	−2.29 (56)*
Sex, *n*(%) boys	19 (68%)	21 (70%)	−0.17 (56)
IQ, *M* (*SD*)	92.92 (11.37)^a^	92.87 (11.48)	−0.02 (52)
Parental education, *M* (*SD*)^b^	4.91 (1.35)	5.02 (1.29)	0.31 (55)
*Clinical characteristics*			
ADHD presentation (C/I/HI/Subthr.)^c^	9/9/8/2	21/5/4/0	9.22 (3)*
Daily ratings of target behaviors, *M* (*SD*)	2.33 (0.97)	2.37 (0.89)	0.16 (56)
ECBI (intensity), *M* (*SD*)	3.53 (0.71)	–^d^	–^d^
SWAN (inattention), *M* (*SD*)	7.89 (8.09)	11.64 (6.92)	1.86 (54)
SWAN (Hyp./Imp.), *M* (*SD*)	9.21 (8.11)	12.89 (7.53)	1.76 (54)
DBDRS (ODD), *M* (*SD*)	6.82 (5.13)	7.53 (5.41)	0.51 (54)
IRS, *M* (*SD*)	3.19 (1.08)	3.53 (1.06)	1.19 (54)
PSOC (self‐efficacy), *M* (*SD*)	3.99 (0.70)	–^d^	–^d^

Chi‐square tests were used to compare groups on sex and ADHD presentation, *t*‐tests were used to compare groups on all other measures. We reverse‐coded the SWAN to be consistent with the other measures.

^a^ *n* = 24, 4 missing.

^b^ Classified according to the Dutch classification system (CBS, 2016): 1 = no education completed up to 8 = postgraduate education.

^c^ ADHD presentation C = combined, I = inattentive, HI = hyperactive/impulsive, Subthr. = subthreshold.

^d^ Not applicable.

* *p* < .05.

### Feasibility

#### Feasibility of the program

In total, 14.2% of parents dropped out of treatment. Reasons for dropout were severe problems of the child that needed other treatment immediately (*n* = 2), or other problems in the family that required attention (*n* = 2). Parents' satisfaction was high, with a mean score of 4.4 (scale 1–5; data of five parents were missing; see Figure S1a for details). Therapists were very satisfied (*M =* 4.8; scale 1–5; see Figure S1b).

#### Feasibility of study procedures

**Figure 2:**
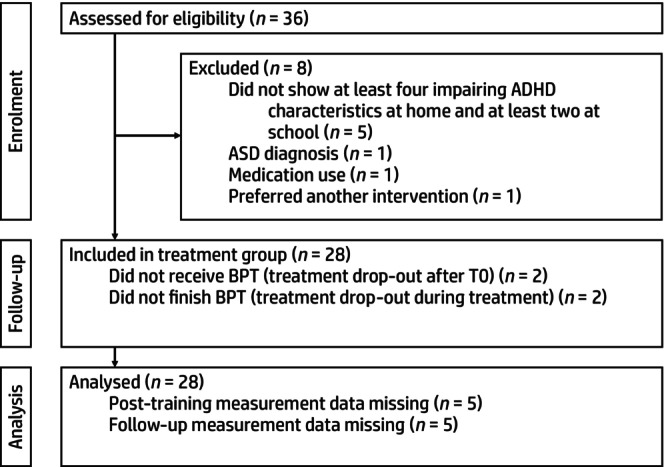
CONSORT flow diagram

We recruited 28 participants in 19 months (average of 1.5 per month, see Figure 2 for the CONSORT flow diagram). Of the four parents who dropped out of treatment, three also failed to complete all measurements (10.7%). Response and completion rates on questionnaires were 100% at T0, 82% and 100%, respectively, at T2, and 82% and 99%, respectively, at T3. For daily measurements, response rates were 100% at T0 and 98% at T1, T2, and T3.

Parents judged the outcome measures as acceptable: the number and length of daily measurements and online questionnaires were “exactly enough” (acceptability score *M =* 3.3; scale ranging from 1 [too few/short] to 5 [too much/long]), measurements were not very burdensome for parents (burden score *M =* 2.2; scale ranging from 1 [not burdensome] to 4 [very burdensome]), parents agreed that completing the questionnaires was doable (*M =* 3.6; scale ranging from 1 [strongly disagree] to 5 [strongly agree]). On average, parents needed 23 min to complete all measures. See Figure S1c–e for detailed information on feasibility/acceptability analyses. Treatment fidelity was high: 96% as reported by self‐evaluation forms from therapists and 97% as reported by independent coders who scored audio‐recordings of a random sample of 20% of all sessions.

### Pre–post changes within and between groups

Table 3 shows all within‐ and between‐group outcomes. For all within‐group analyses, the random intercepts at the therapist level (level 3) did not significantly improve the fit of the models. All models were therefore reduced to two levels. To take baseline differences between groups into account, we included age and ADHD presentation as fixed factors in all between‐group analyses. The number of days between T0 and T3 was not associated with the changes on our primary outcome at T1, T2 and T3 (*r* = −.03, *r* = .25, *r* = .15, *p*'s > .25).

**Table 3:** Within‐ and between‐group effects

	T0–T1		T0–T2		T0–T3	
	*t* (df)	*d*	*t* (df)	*d*	*t* (df)	*d*
*Daily ratings of target behaviors*						
Within‐group effects	−4.49 (71.81)	.68***	−4.71 (72.26)	.75***	−4.89 (72.26)	.77***
Between‐group effects	−3.40 (108.78)	.46***	−3.44 (109.03)	.48***		
*ECBI (intensity)*						
Within‐group effects			−1.07 (47.06)	.16	−2.54 (47.06)	.37*
Between‐group effects						
*SWAN (inattention)*						
Within‐group effects			.04 (49.09)	.01	−1.38 (49.09)	.26
Between‐group effects			.51 (53.01)	.17		
*SWAN (hyperactivity/impulsivity)*						
Within‐group effects			−.77 (48.37)	.16	−1.80 (48.37)	.38
Between‐group effects			−.50 (55.89)	.21		
*DBDRS (ODD)*						
Within‐group effects			−.01 (47.40)	.00	−1.02 (47.40)	.17
Between‐group effects			.75 (52.58)	.06		
*IRS*						
Within‐group effects			−1.97 (47.51)	.34	−3.89 (47.51)	.67***
Between‐group effects			−.92 (51.92)	.39		
*PSOC (self‐efficacy)*						
Within‐group effects			.98 (47.76)	.18	1.37 (47.76)	.25
Between‐group effects						

We reverse‐coded the SWAN to be consistent with the other measures. For within‐group comparisons, negative scores indicate a decrease in child behaviors/symptoms or parents' self‐efficacy. For between‐group comparisons, negative scores indicate improvement of the treatment group compared to the historical control group. Empty cells: not administered in the control group at that timepoint.

*p < .05, **p < .01, ***p < .001.

## Discussion

In this pilot study, which was conducted in preparation for an RCT, our first objective was to investigate the feasibility of a newly developed brief BPT program for children with ADHD characteristics and to examine the feasibility of study procedures. The feasibility of the program was good. Dropout was relatively low (14.2%) compared to traditional BPT programs (i.e., 26% [Chacko et al., 2016]), and parents' and therapists' satisfaction was high. The feasibility of study procedures was acceptable, except recruitment rates.

Our second objective was to assess pre–post changes. Directly after treatment, we observed improvements in children's daily‐rated problem behaviors in the treatment group compared to the historical control group. Within the treatment group, effect sizes further increased at follow‐up. Regarding daily‐rated problem behaviors, within‐group effect sizes were similar to those in our microtrial (Hornstra et al., 2021).

We found no significant short‐term between‐ or within‐group effects on secondary outcomes, which might suggest that the benefits do not extend beyond the targeted behaviors. However, at follow‐up (3–8 weeks after the booster session), children's behavioral problems and impairment significantly decreased compared to posttreatment. Therefore, further research is necessary to investigate beneficial effects on secondary outcomes as well as longer‐term effects.

Due to the lack of a randomly assigned control group, we cannot rule out that other factors such as the natural course of children's development were associated with the improvements. Nevertheless, the promising within‐group effect sizes on daily‐rated problem behaviors and the feasibility of program and study procedures encouraged us to start an RCT of the program (ClinicalTrials.gov: NCT05591820). In this study, we investigate the short‐ and longer‐term effectiveness of the program, relative to care‐as‐usual. To ensure feasible recruitment, this RCT is being carried out across multiple centres.

The current study had some important limitations. First, we used a historical control group. Significant differences between groups should therefore be interpreted cautiously, especially given the differences in age and ADHD presentations between groups (Cuffe, 2011). Also two children with subthreshold ADHD were included in the current study, whereas in the previous study, these participants would have been excluded. Second, although properly powered for our within‐group analyses, our sample size was small and contained some missing data, limiting the generalizability of our findings. Third, all findings depend on parent‐reported measures, which may be biased. However, they are informative for clinical care decisions. Fourth, our feasibility measures had no predetermined success criteria. Nevertheless, they provided relevant information on consumer experiences and potential barriers we can anticipate in future studies.

To conclude, this pilot has informed further research on brief BPT. If the program is effective in future research, this would be beneficial for clinical practice. Brief BPT could ultimately reduce barriers for parents in clinical practice and thereby enhance the accessibility of BPT (Dekkers, Groenman, et al., 2022).

## Conflict of interest statement

BH and SO were involved in the development of the brief behavioral parent training that was investigated in this study. SO has co‐developed planning‐focused and solution‐focused treatment and other behavioral treatments but has no financial interest in any of these. She was an advisor of the Dutch ADHD guideline groups and is a member of a working group on ADHD of the Superior Health Council of Belgium. BdH has received royalties as one of the editors of *Sociaal Onhandig* (published by Van Gorcum), a Dutch book for parents that can be used in parent training. She has been involved in the development and evaluation of several parent and teacher training programs, without financial interests; she has been a member of Dutch ADHD guideline and practice standard groups. The other authors report no conflict of interest.

## Funding information

There was no external funding for this study.

## Author contributions

Conceptualization: MN, BH, TD; Data collection: MN, RH; Data analysis: RD; Supervision data analysis: AG; Project supervision: BH, TD; Writing: MN, RD; Reviewing and editing: AG, SO, RH, BH, TD. MN and TD have full access to all data in the study and take responsibility for the integrity of the data and the accuracy of the data analysis.

## Ethical information

All participating parents provided informed consent. The study was preregistered (ClinicalTrials.gov ID: NCT05452954) and the Medical Ethics Review Board of the University Medical Centre Groningen (UMCG) approved that it did not meet the conditions of the Medical Research Involving Human Subjects Act (WMO). The study complied with the declaration of Helsinki (1964) and its later revisions.

## Supporting information


**Appendix S1.** Measures.
**Appendix S2.** Means and standard deviations of outcome measures.
**Figure S1.** Feasibility/acceptability analyses. (a) Parent satisfaction with the brief parent training. (b) Therapist satisfaction with the brief parent training. (c) Parent satisfaction with the measurements. (d) Parent satisfaction with the measurements. (e) Parent satisfaction with the measurements.
**Figure S2.** Means and standard deviations on the primary outcome per group per timepoint.
**Table S1.** Means and standard deviations on the primary and secondary outcomes per group per timepoint.
**Table S2.** CONSORT checklist.

## Data Availability

Data available upon request due to privacy/ethical restrictions.
