# Supplementary material for: Innovations in Practice: Brief behavioral parent training for children with impairing ADHD characteristics – a pilot study
Source: Child Adolesc Ment Health. 2024 Dec 7;30(1):83–8. doi: 10.1111/camh.12743 (PMC11754698; doi:10.1111/camh.12743)
Supplement: Supplementary file 1 — Appendix S1. Measures. Appendix S2. Means and standard deviations of outcome measures. Figure S1. Feasibility/acceptability analyses. (a) Parent satisfaction with the brief parent training. (b) Therapist satisfaction with the brief parent training. (c) Parent satisfaction with the measurements. (d) Parent satisfaction with the measurements. (e) Parent satisfaction with the measurements. Figure S2. Means and standard deviations on the primary outcome per group per timepoint. Table S1. Means and standard deviations on the primary and secondary outcomes per group per timepoint. Table S2. CONSORT checklist. [file CAMH-30-83-s001.zip › Figure S1e Parent Satisfaction with the Measurements 3.docx]

**Figure S1e.** *Parent Satisfaction with the Measurements*


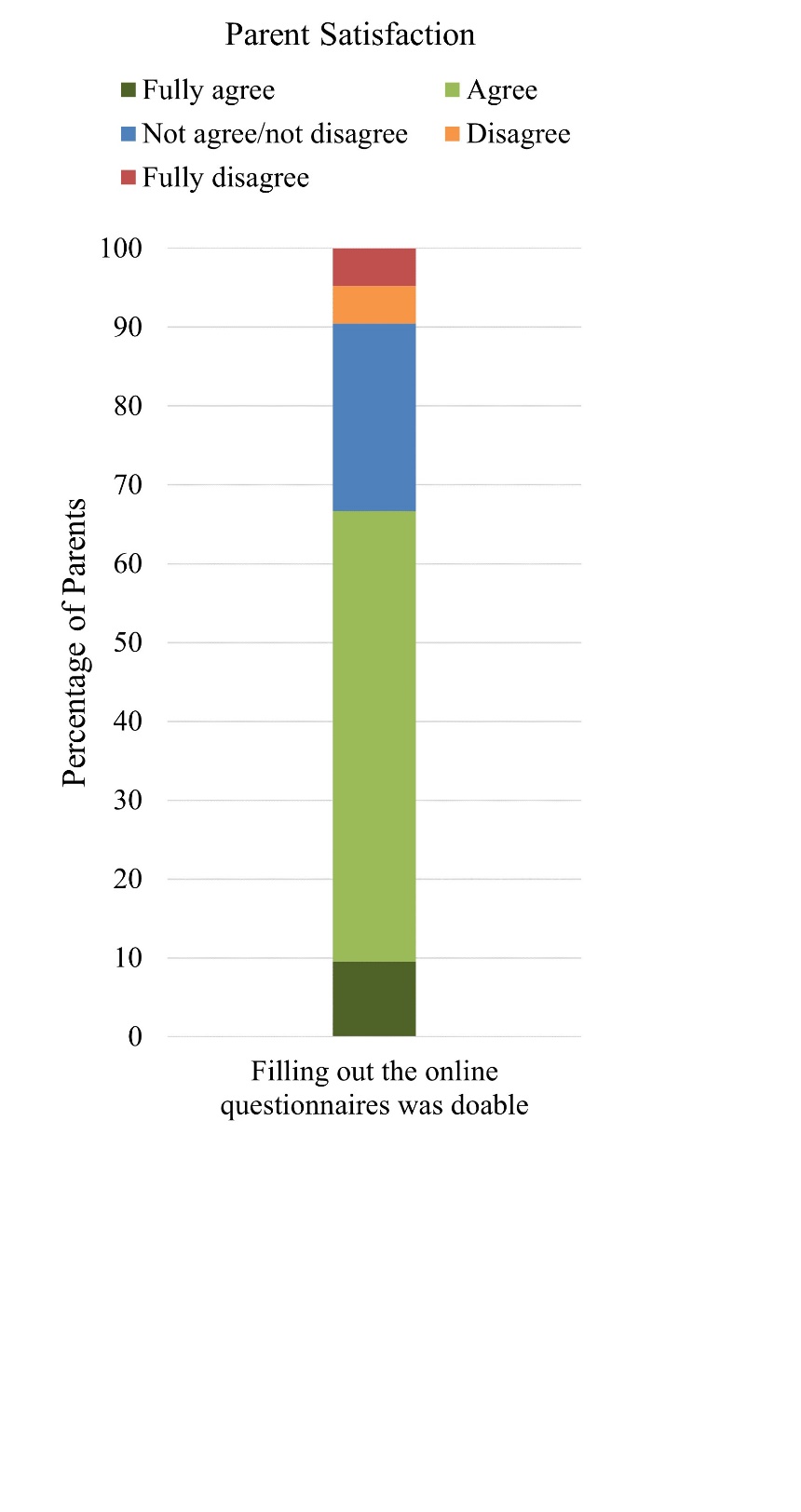


*Note. N* = 21. The answers of seven parents (25%) were missing.
