# Supplementary material for: Innovations in Practice: Brief behavioral parent training for children with impairing ADHD characteristics – a pilot study
Source: Child Adolesc Ment Health. 2024 Dec 7;30(1):83–8. doi: 10.1111/camh.12743 (PMC11754698; doi:10.1111/camh.12743)
Supplement: Supplementary file 1 — Appendix S1. Measures. Appendix S2. Means and standard deviations of outcome measures. Figure S1. Feasibility/acceptability analyses. (a) Parent satisfaction with the brief parent training. (b) Therapist satisfaction with the brief parent training. (c) Parent satisfaction with the measurements. (d) Parent satisfaction with the measurements. (e) Parent satisfaction with the measurements. Figure S2. Means and standard deviations on the primary outcome per group per timepoint. Table S1. Means and standard deviations on the primary and secondary outcomes per group per timepoint. Table S2. CONSORT checklist. [file CAMH-30-83-s001.zip › Nijboer et al. 37.R3 - Supporting Information [2].docx]

**Appendix S1.** Measures.

**Participant Characteristics**

***Intelligence***

Participants were included if children’s IQ was higher than 70. IQ-scores were based on the Wechsler Intelligence Scale for Children-V (Dutch edition; WISC-V-NL; Wechsler, 2018) or the Wechsler Preschool and Primary Scale of Intelligence-III (Dutch edition; WPSSI-III-NL; Hurks & Hendriksen, 2020), and were derived from the medical file. Of four participants an IQ-score was missing but their IQ was estimated by their clinician as higher than 70.

***Parents’ educational level***

Parents’ educational level (average of both parents) was classified according to the Dutch classification system (CBS, 2016): 1 = no education completed, 2 = early childhood education, 3 = primary education, 4 = lower secondary education, 5 = upper secondary education, 6 = undergraduate school, 7 = graduate school, 8 = post-graduate education.

***ADHD presentation***

ADHD presentation of the children in the treatment group was derived from the medical file and was assessed with Parent Interview for Child Symptoms (PICS; Schachar, Ickowicz & Sugarman, 2000) and Teacher Telephone Interview (TTI; Tannock et al., 2002). In the historical control group, ADHD presentation was measured with the Diagnostic Interview Schedule for Children (DISC; Shaffer et al., 2000). All are (semi-)structured interviews to assess symptoms and criteria according to the DSM. We identified four presentations: combined, inattentive, hyperactive/impulsive, subthreshold (i.e. 4 or 5 impairing symptoms of inattentiveness, hyperactivity/impulsivity, or both).

**Feasibility of treatment program**

We recorded treatment drop-out, parent satisfaction and therapist satisfaction.

***Treatment drop-out***

Percentages of families dropping out of treatment before finishing the booster session were reported.

***Parent satisfaction***

Parent satisfaction and acceptability of the intervention was measured using an evaluation questionnaire containing 25 items (23 Likert scale items and two open questions) about parental satisfaction and usefulness of training parts, the ease with which parents could combine the training with daily life, the use of learned skills, the perceived influence on their child’s behaviour, perceived helpfulness of the training, and whether parents would recommend the training to other parents.

The parent satisfaction questionnaire used in this study is based on the Parent Satisfaction Questionnaire (Bearss et al., 2013) and the Therapy Attitude Inventory (Eyberg, 1993; Eyberg & Johnson, 1974). Ratings were made on a 5-point scale, ranging from 1 ‘totally disagree’ to 5 ‘totally agree’. The mean satisfaction score over 23 items was reported as well as the percentage on each answer category per item.

***Therapist satisfaction***

Therapists’ satisfaction was measured using a short self-developed evaluation questionnaire (18 items) on acceptability and usefulness of the intervention according to therapists. Ratings were made on a 5-point Likert scale, with answers ranging from 1 ‘too few/too short/strongly disagree’ to 5 ‘too much/too long/strongly agree’. Based on the items a mean satisfaction score was calculated.

**Feasibility of study procedures**

We analysed recruitment rates, study drop-out, parent-rated acceptability of measurements, response and completion rates on outcome measures, and treatment fidelity.

***Recruitment rates***

The total number of recruited participants was reported, including an average per month. Also the number of participants meeting the inclusion criteria but not willing to participate included was reported, as well as the reasons.

***Study drop-out***

Percentages of families dropping out the study before finishing follow-up measurement were reported.

***Response and completion rates***

The percentage of parents who responded on all timepoints were reported, as well as the percentage of completed measures at all timepoints.

***Parent-rated acceptability of outcome measures***

Parent-rated acceptability of primary and secondary outcome measures was measured using seven self-developed evaluation questions on frequency and duration of measurements (six Likert scale items, one open question on minutes needed to complete questionnaires). On six items ratings were made on a 5-point Likert scale, with answers ranging from 1 ‘too few/too short/strongly disagree’ to 5 ‘too much/too long/strongly agree’. Mean scores were reported on acceptability of number and length of daily measurements and online questionnaires (3 items), burden of measurements (2 items) and ease of completing the questionnaires (1 item). An average number of minutes needed to complete the questionnaires was reported.

***Treatment fidelity***

Therapists were asked to make audio records of each session and to complete a treatment fidelity checklist after each session. The percentage of addressed session items was checked in two ways: through the session-forms from the therapists, and by an independent rater scoring the selected audiotapes (random sample of 20% of all sessions).

**Pre-post changes**

***Daily Ratings of Target Behaviours (Primary Outcome)***

Parents identified behaviours that the child displayed daily at home from a list of target behaviours (Hornstra et al., 2021; see also Van den Hoofdakker et al., 2007). This list contains 29 problem behaviours (e.g., disobedience, whining, not waiting their turn). For each item, parents indicated whether the behaviour occurred daily (yes/no), and, for the items scored as yes, how severely the behaviour occurred on a 5-point Likert scale ranging from 1 (not severe) to 5 (extremely severe). With a researcher, parents then selected four of the daily occurring behaviours they wanted to target in the brief parent training and specified in which situations the problem behaviours occurred. During each measurement occasion, through short phone calls on five consecutive schooldays, parents indicated whether each of the four selected behaviours occurred that day (yes/no) and if so, how severely on a 5-point Likert scale.

***ECBI (Intensity Scale)***

Child disruptive behaviour was measured with the Intensity subscale of the Dutch version of the Eyberg Child Behaviour Inventory (Eyberg & Ross, 1978; Eyberg & Pincus, 1999). The ECBI is a 36-item questionnaire for parents of children aged 2 to 16. The Intensity subscale measures the frequency of specific problem behaviour on a 7-point Likert scale from 1 (never) to 7 (always) (Weeland et al., 2018). Examples of items include “Refuses to go to bed on time”, “Has temper tantrums”, and “Is easily distracted”. The convergent and divergent validity of the ECBI are good (Abrahamse et al., 2015).

***SWAN (Inattention and Hyperactivity/Impulsivity Scales)***

Child ADHD symptoms were measured with the Strengths and Weaknesses of ADHD symptoms and Normal behaviour rating scale (SWAN). The convergent and discriminant validity of the SWAN are well established (Swanson, Schuck & Porter, 2012). The SWAN consists of the Inattention subscale (9 items) and the Hyperactivity/Impulsivity subscale (9 items). Each item can be scored on a 7-point Likert scale ranging from “far below average” (1) to “far above average” (7). Examples of items include “Gives close attention to detail and avoids careless mistakes” and “Stays seated (when required by class rules or social conventions)”. We reverse coded the SWAN to be consistent with the other outcome measures.

***DBDRS (ODD subscale)***

Children’s ODD symptoms were measured with the ODD subscale of the Disruptive Behaviour Disorder Rating Scale (DBDRS; Pelham et al., 1992; Oosterlaan et al., 2008). The ODD subscale contains eight items, on which parents rate the occurrence of child behaviours on a 4-point Likert scale, ranging from not at all (1) to very much (4). Examples of items include “often argues with adults” and “is often spiteful or vindictive”. The ODD subscale has good construct validity and internal consistency (α=.88; Oosterlaan et al., 2008).

***IRS***

Impairment of the child was measured with the Impairment Rating Scale (IRS), of which the validity has been demonstrated (Fabiano, 2006). The IRS measures impairment on seven domains, including relationships with peers, siblings, and parents, family functioning, academic progress, self-esteem, and overall impairment on a 7-point Likert scale from no problem (0) to extreme problem (6).

***PSOC (Efficacy subscale)***

Parents’ sense of parenting competence was measured with the Efficacy subscale of the Parenting Sense of Competence Scale (PSOC; Johnston & Mash, 1989). The Efficacy subscale contains eight items, on which parents rate their capability level and problem-solving ability regarding their parental role on a 6-point Likert scale, ranging from strongly disagree (1) to strongly agree (6). Examples of items include “Being a parent is manageable, and any problems are easily solved” and “I honestly believe I have all the skills necessary to be a good parent to my child”. The internal consistency (α=.76) of the Efficacy subscale has been established (Johnston & Mash, 1989).

**Figure S1.** Feasibility/Acceptability Analyses.

**Figure S1a.** Parent Satisfaction with the Brief Parent Training.

[insert Figure S1a here]

**Figure S1b.** Therapist Satisfaction with the Brief Parent Training.

[insert Figure S1b here]

**Figure S1c.** Parent Satisfaction with the Measurements.

[insert Figure S1c here]

**Figure S1d.** Parent Satisfaction with the Measurements.

[insert Figure S1d here]

**Figure S1e.** Parent Satisfaction with the Measurements.

[insert Figure S1e here]

**Appendix S2.** Means and Standard Deviations of outcome measures.

**Table S1.** *Means and Standard Deviations on the Primary and Secondary Outcomes per Group per Timepoint.*

|  | T0  *M(SD)* | T1  *M(SD)* | T2  *M(SD)* | T3  *M(SD)* |
| --- | --- | --- | --- | --- |
| Daily ratings of target behaviours |  |  |  |  |
| Treatment group | 2.33(.97) | 1.58(1.01) | 1.50(.78) | 1.47(.80) |
| Historical control group | 2.37(.89) | 2.32(.86) | 2.29(.92) | - |
| ECBI (intensity) |  |  |  |  |
| Treatment group | 3.53(.71) | - | 3.43(.84) | 3.29(.67) |
| Historical control group | - | - | - | - |
| SWAN (inattention) |  |  |  |  |
| Treatment group | 7.89(8.09) | - | 8.35(10.65) | 5.09(10.93) |
| Historical control group | 11.64(6.92) | - | 10.72(7.47) | - |
| SWAN (hyperactivity/impulsivity) |  |  |  |  |
| Treatment group | 9.21(8.11) | - | 7.48(10.85) | 5.30(12.08) |
| Historical control group | 12.89(7.53) | - | 12.83(7.28) | - |
| DBDRS (ODD) |  |  |  |  |
| Treatment group | 6.82(5.14) | - | 6.78(4.94) | 6.26(5.19) |
| Historical control group | 7.54(5.41) | - | 6.83(4.55) | - |
| IRS |  |  |  |  |
| Treatment group | 3.19(1.08) | - | 2.79(1.24) | 2.35(1.33) |
| Historical control group | 3.53(1.06) | - | 3.40(1.13) | - |
| PSOC (self-efficacy) |  |  |  |  |
| Treatment group | 3.99(.70) | - | 4.15(.99) | 4.21(.91) |
| Historical control group | - | - | - | - |

ECBI = Eyberg Eyberg Child Behaviour Inventory; SWAN = Strengths and Weaknesses of ADHD symptoms and Normal behaviour rating scale; DBDRS (ODD) = Disruptive Behaviour Disorder Rating Scale (Oppositional Defiant Disorder subscale); IRS = Impairment Rating Scale; PSOC = Parenting Sense of Competence Scale.

*Note.* We reverse coded the SWAN to be consistent with the other outcome measures.

**Figure S2.** *Means and Standard Deviations on the Primary Outcome per Group per Timepoint.*

[insert Figure S2 here]

Table S2. *CONSORT 2010 checklist of information to include when reporting a pilot or feasibility trial*.*

| Section/Topic | Item No | Checklist item | Reported on page No |
| --- | --- | --- | --- |
| Title and abstract | | | |
|  | 1a | Identification as a pilot or feasibility randomised trial in the title | 1 |
|  | 1b | Structured summary of pilot trial design, methods, results, and conclusions (for specific guidance see CONSORT abstract extension for pilot trials) | 1-2 |
| Introduction | | | |
| Background and objectives | 2a | Scientific background and explanation of rationale for future definitive trial, and reasons for randomised pilot trial | 4 |
|  | 2b | Specific objectives or research questions for pilot trial | 4 |
| Methods | | | |
| Trial design | 3a | Description of pilot trial design (such as parallel, factorial) including allocation ratio | 6 |
|  | 3b | Important changes to methods after pilot trial commencement (such as eligibility criteria), with reasons | N/A |
| Participants | 4a | Eligibility criteria for participants | 5 |
|  | 4b | Settings and locations where the data were collected | 5 |
|  | 4c | How participants were identified and consented | 5 |
| Interventions | 5 | The interventions for each group with sufficient details to allow replication, including how and when they were actually administered | 6-7 |
| Outcomes | 6a | Completely defined prespecified assessments or measurements to address each pilot trial objective specified in 2b, including how and when they were assessed | 8-9, Appendix S1 |
|  | 6b | Any changes to pilot trial assessments or measurements after the pilot trial commenced, with reasons | N/A |
|  | 6c | If applicable, prespecified criteria used to judge whether, or how, to proceed with future definitive trial | N/A |
| Sample size | 7a | Rationale for numbers in the pilot trial | 9 |
|  | 7b | When applicable, explanation of any interim analyses and stopping guidelines | N/A |
| Randomisation: |  |  |  |
| Sequence  generation | 8a | Method used to generate the random allocation sequence | N/A |
|  | 8b | Type of randomisation(s); details of any restriction (such as blocking and block size) | N/A |
| Allocation  concealment  mechanism | 9 | Mechanism used to implement the random allocation sequence (such as sequentially numbered containers), describing any steps taken to conceal the sequence until interventions were assigned | N/A |
| Implementation | 10 | Who generated the random allocation sequence, who enrolled participants, and who assigned participants to interventions | N/A |
| Blinding | 11a | If done, who was blinded after assignment to interventions (for example, participants, care providers, those assessing outcomes) and how | N/A |
|  | 11b | If relevant, description of the similarity of interventions | N/A |
| Statistical methods | 12 | Methods used to address each pilot trial objective whether qualitative or quantitative | 9 |
| Results | | | |
| Participant flow (a diagram is strongly recommended) | 13a | For each group, the numbers of participants who were approached and/or assessed for eligibility, randomly assigned, received intended treatment, and were assessed for each objective | 11 |
|  | 13b | For each group, losses and exclusions after randomisation, together with reasons | 11 |
| Recruitment | 14a | Dates defining the periods of recruitment and follow-up | 4 |
|  | 14b | Why the pilot trial ended or was stopped | 11/ N/A |
| Baseline data | 15 | A table showing baseline demographic and clinical characteristics for each group | 10 |
| Numbers analysed | 16 | For each objective, number of participants (denominator) included in each analysis. If relevant, these numbers  should be by randomised group | 10-11, Figure S1 |
| Outcomes and estimation | 17 | For each objective, results including expressions of uncertainty (such as 95% confidence interval) for any  estimates. If relevant, these results should be by randomised group | Not assessed |
| Ancillary analyses | 18 | Results of any other analyses performed that could be used to inform the future definitive trial | 10-11 |
| Harms | 19 | All important harms or unintended effects in each group (for specific guidance see CONSORT for harms) | Not assessed |
|  | 19a | If relevant, other important unintended consequences | Not assessed |
| Discussion | | | |
| Limitations | 20 | Pilot trial limitations, addressing sources of potential bias and remaining uncertainty about feasibility | 15 |
| Generalisability | 21 | Generalisability (applicability) of pilot trial methods and findings to future definitive trial and other studies | 14 |
| Interpretation | 22 | Interpretation consistent with pilot trial objectives and findings, balancing potential benefits and harms, and  considering other relevant evidence | 14-15 |
|  | 22a | Implications for progression from pilot to future definitive trial, including any proposed amendments | 14 |
| Other information | | |  |
| Registration | 23 | Registration number for pilot trial and name of trial registry | 6 |
| Protocol | 24 | Where the pilot trial protocol can be accessed, if available | 6 |
| Funding | 25 | Sources of funding and other support (such as supply of drugs), role of funders | 15-16 |
|  | 26 | Ethical approval or approval by research review committee, confirmed with reference number | 6 |

Citation: Eldridge SM, Chan CL, Campbell MJ, Bond CM, Hopewell S, Thabane L, et al. CONSORT 2010 statement: extension to randomised pilot and feasibility trials. BMJ. 2016;355. This is an Open Access article distributed in accordance with the terms of the Creative Commons Attribution (CC BY 3.0) license (<http://creativecommons.org/licenses/by/3.0/>), which permits others to distribute, remix, adapt and build upon this work, for commercial use, provided the original work is properly cited.

*We strongly recommend reading this statement in conjunction with the CONSORT 2010, extension to randomised pilot and feasibility trials, Explanation and Elaboration for important clarifications on all the items. If relevant, we also recommend reading CONSORT extensions for cluster randomised trials, non-inferiority and equivalence trials, non-pharmacological treatments, herbal interventions, and pragmatic trials. Additional extensions are forthcoming: for those and for up-to-date references relevant to this checklist, see [www.consort-statement.org](http://www.consort-statement.org)

**References**

Abrahamse, M. E., Junger, M., Leijten, P. H. O., Lindeboom, R., Boer, F., & Lindauer, R. J. L. (2015). Psychometric Properties of the Dutch Eyberg Child Behaviour Inventory (ECBI) in a Community Sample and a Multi-Ethnic Clinical Sample. *Journal of Psychopathology and Behavioural Assessment*, *37*(4), 679–691. https://doi.org/10.1007/s10862-015-9482-1

Bearss, K., Johnson, C., Handen, B., Smith, T., & Scahill, L. (2013). A Pilot Study of Parent Training in Young Children with Autism Spectrum Disorders and Disruptive Behaviour. *Journal of Autism and Developmental Disorders*, *43*(4), 829–840. https://doi.org/10.1007/s10803-012-1624-7

CBS. (2016). *Standaard onderwijsindeling*. Centraal Bureau voor de Statistiek.

Eyberg, S. M. (1993). *Consumer satisfaction measures for assessing parent training programs* (L. VandeCreek, S. Knapp, & T. L. Jackson, Eds.; pp. 377–382). Professional Resource Press/Professional Resource Exchange.

Eyberg, S. M., & Johnson, S. M. (1974). Multiple assessment of behaviour modification with families: Effects of contingency contracting and order of treated problems. *Journal of Consulting and Clinical Psychology*, *42*(4), 594–606. https://doi.org/10.1037/h0036723

Eyberg, S. M., & Pincus, D. (1999). *ECBI & SESBI-R: Eyberg child behaviour inventory and sutter-eyberg student behaviour inventory-revised: Professional manual.* Psychological Assessment Resources.

Eyberg, S. M., & Ross, A. W. (1978). Assessment of child behaviour problems: The validation of a new inventory. *Journal of Clinical Child Psychology*, *7*(2), 113–116. https://doi.org/10.1080/15374417809532835

Eldridge S.M., Chan C.L., Campbell M.J., Bond C.M., Hopewell S., Thabane L., et al. CONSORT 2010 statement: extension to randomised pilot and feasibility trials. BMJ. 2016;355.

Fabiano, G. A., Pelham, Jr. , W. E., Waschbusch, D. A., Gnagy, E. M., Lahey, B. B., Chronis, A. M., Onyango, A. N., Kipp, H., Lopez-Williams, A., & Burrows-MacLean, L. (2006). A Practical Measure of Impairment: Psychometric Properties of the Impairment Rating Scale in Samples of Children With Attention Deficit Hyperactivity Disorder and Two School-Based Samples. *Journal of Clinical Child & Adolescent Psychology*, *35*(3), 369–385. https://doi.org/10.1207/s15374424jccp3503_3

Hornstra, R., van der Oord, S., Staff, A. I., Hoekstra, P. J., Oosterlaan, J., van der Veen-Mulders, L., Luman, M., & van den Hoofdakker, B. J. (2021). Which Techniques Work in Behavioural Parent Training for Children with ADHD? A Randomized Controlled Microtrial. *Journal of Clinical Child and Adolescent Psychology*, *50*(6), 888–903. https://doi.org/10.1080/15374416.2021.1955368

Hurks, P., & Hendriksen, J. (2020). *WPPSI-IV-NL Wechsler Preschool and Primary Scale of Intelligence*. Pearson.

Johnston, C., & Mash, E. J. (1989). A Measure of Parenting Satisfaction and Efficacy. *Journal of Clinical Child Psychology*, *18*(2), 167–175. https://doi.org/10.1207/s15374424jccp1802_8

Oosterlaan, J., Baeyens, D., Scheres, A., Antrop, I., Roeyers, H., & Sergeant, J. (2008). *VvGK6-16: Vragenlijst voor gedragsproblemen bij kinderen 6 tot en met 16 jaar.* . Pearson.

Pelham, W. E., Gnagy, E. M., Greenslade, K. E., & Milich, R. (1992). Teacher Ratings of DSM-III-R Symptoms for the Disruptive Behaviour Disorders. *Journal of the American Academy of Child & Adolescent Psychiatry*, *31*(2), 210–218. https://doi.org/10.1097/00004583-199203000-00006

Schachar, R., Ickowicz, A., & Sugarman, R. (2000). *Parent interview for child symptoms (PICS‐4): Revised for DSM‐IV (1995)*. The Hospital for Sick Children Department of Psychiatry.

Shaffer, D., Fisher, P., Lucas, C. P., Dulcan, M. K., & Schwab-Stone, M. E. (2000). NIMH Diagnostic Interview Schedule for Children Version IV (NIMH DISC-IV): Description, Differences From Previous Versions, and Reliability of Some Common Diagnoses. *Journal of the American Academy of Child & Adolescent Psychiatry*, *39*(1), 28–38. https://doi.org/10.1097/00004583-200001000-00014

Swanson, J. M., Schuck, S., & Porter, M. (2012). Categorical and dimensional definitions and evaluations of symptoms of ADHD: History of the SNAP and the SWAN rating scales. *The International Journal of Educational and Psychological Assessment*, *10*(11), 21–70.

Tannock, R., Hum, M., Masellis, M., Humphries, T., & Schachar, R. (2002). *Teacher telephone interview for children’s academic performance, attention, behaviour and learning: DSM‐IV Version (TTI‐IV)*. The Hospital for Sick Children.

Van den Hoofdakker, B. J., Van der Veen-Mulders, L., Sytema, S., Emmelkamp, P. M. G., Minderaa, R. B., & Nauta, M. H. (2007). Effectiveness of Behavioural Parent Training for Children With ADHD in Routine Clinical Practice: A Randomized Controlled Study. *Journal of the American Academy of Child & Adolescent Psychiatry*, *46*(10), 1263–1271. https://doi.org/10.1097/chi.0b013e3181354bc2

Wechsler, D. (2018). *WISC-V-NL. Wechsler Intelligence Scale for Children - Fifth Edition - Nederlandse bewerking*. Pearson.

Weeland, J., van Aar, J., & Overbeek, G. (2018). Dutch Norms for the Eyberg Child Behaviour Inventory: Comparisons with other Western Countries. *Journal of Psychopathology and Behavioural Assessment*, *40*(2), 224–234. https://doi.org/10.1007/s10862-017-9639-1
